# Uptake and Metabolization of Serotonin by Granulosa Cells Form a Functional Barrier in the Mouse Ovary

**DOI:** 10.3390/ijms232314828

**Published:** 2022-11-27

**Authors:** Nina M. Alyoshina, Maria D. Tkachenko, Lyudmila A. Malchenko, Yuri B. Shmukler, Denis A. Nikishin

**Affiliations:** 1 N.K. Koltzov Institute of Developmental Biology, Russian Academy of Sciences, Vavilova Street, 26, 119334 Moscow, Russia; 2 Faculty of Biology, Lomonosov Moscow State University, Leninskie Gory, 1, bld. 12, 119991 Moscow, Russia

**Keywords:** serotonin, ovary, oocyte, granulosa cells, SERT, MAO, blood-follicle-barrier, pargyline, fluoxetine

## Abstract

Serotonin (5-HT) plays an essential role in regulating female reproductive function in many animals. 5-HT accumulates in the mammalian ovary with the involvement of membrane serotonin transporter SERT and is functionally active in the oocytes of growing follicles, but shows almost no activity in follicular cells. In this study, we clarified the interplay between 5-HT membrane transport and its degradation by monoamine oxidase (MAO) in the mammalian ovary. Using pharmacologic agents and immunohistochemical staining of the cryosections of ovaries after serotonin administration in vitro, we demonstrated the activity of transport and degradation systems in ovarian follicles. The MAO inhibitor pargyline increased serotonin accumulation in the granulosa cells of growing follicles, indicating the activity of both serotonin uptake and degradation by MAO in these cells. The activity of MAO and the specificity of the membrane transport of serotonin was confirmed in primary granulosa cell culture treated with pargyline and fluoxetine. Moreover, the accumulation of serotonin is more effective in the denuded oocytes and occurs at lower concentrations than in the oocytes within the follicles. This confirms that the activity of SERT and MAO in the granulosa cells surrounding the oocytes impedes the accumulation of serotonin in the oocytes and forms a functional barrier to serotonin.

## 1. Introduction

Although serotonin (5-hydroxitryptamine, 5-HT) is primarily considered a neurotransmitter, only 2% of it functions in neurons [1]. Almost all 5-HT is distributed in the periphery, mainly in the gut and blood platelets [2], and is involved in the regulation of a number of physiological processes [1]. 5-HT also plays an important role in the female reproductive system, as its conserved function is to modulate egg maturation, ovulation, and early embryonic development [3,4,5]. The importance of studying 5-HT in the ovaries is related to the widespread use of neurochemical drugs, such as antidepressants. Specific 5-HT reuptake inhibitors (SSRIs) and monoamine oxidase inhibitors (MAOIs) have a direct effect on 5-HT levels in the central nervous system and are commonly prescribed to treat depression. Since these drugs also alter peripheral 5-HT levels [6], it is crucial to understand the functional mechanisms of the ovarian serotonergic system. Quite a few components of the serotonergic system have already been described in the mouse ovary [7]. Previously, we have shown that the main source of 5-HT in the oocytes of growing follicles is membrane uptake driven by the 5-HT-specific transporter SERT (Slc6a4) [8]. However, the opposite process—the degradation mechanism—has not yet been described.

Monoamine oxidases (MAOs) are enzymes that cause the degradation of various molecules with amine origins: amines, amine hormones, and neurotransmitters through oxidative deamination [9]. MAOs are divided into two types: MAO-A, which is responsible for the degradation of 5-HT, norepinephrine, and epinephrine; and MAO-B, which oxidizes phenylethylamine and benzylamine. Dopamine, tyramine, and tryptamine can be degraded by both MAO forms [10]. In the case of antidepressant treatment, most MAOIs show nonspecific effects on both types of MAO [11]. Pargyline is considered an inhibitor of MAO-B, but also increases tissue 5-HT levels [12].

Previously, we showed that the accumulation of 5- HT by SERT is most functionally active in oocytes and shows almost no activity in follicular cells [8]. Although it is known that granulosa cells are directly connected to oocytes via gap junctions, which allows the latter to be supplied with hormones, growth factors, and other small molecules [13], the mechanism by which 5- HT reaches the oocyte was previously unknown. In this study, we clarified the interplay of degradation and transport systems in the growing ovarian follicle of the mouse ovary.

## 2. Results

### 2.1. MAO Shows Its Activity in Granulosa Cells of Growing Follicles

**Figure 1:**
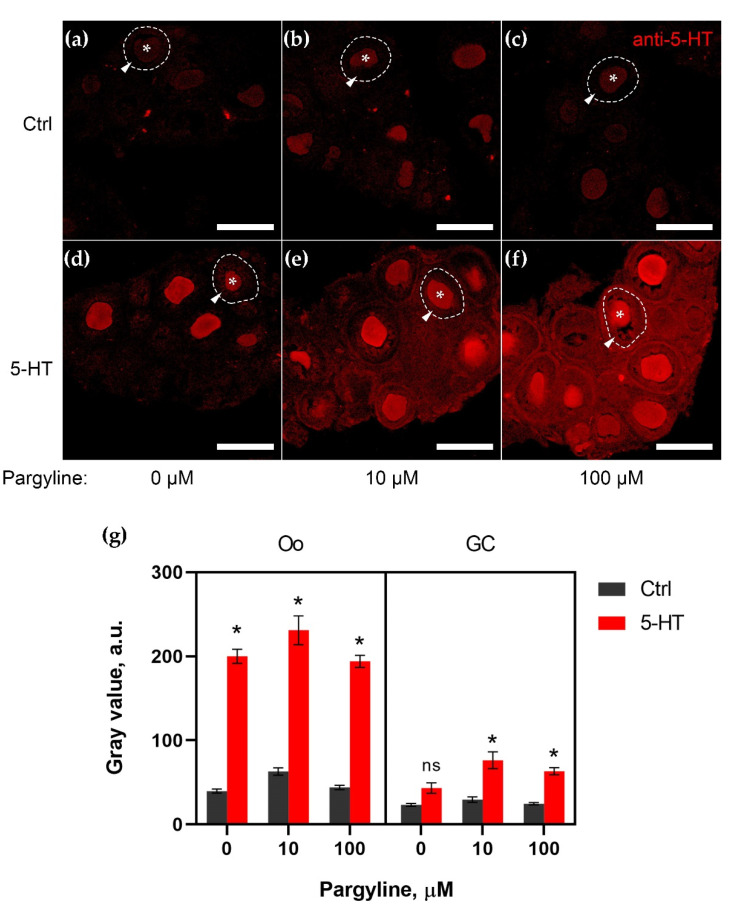
Effect of pargyline on exogenous 5-HT accumulation in the ovary. (**a**–**g**) Immunohistochemical detection of 5-HT in ovarian tissue after a two-hour incubation with 1 μM 5-HT prepared by confocal laser scanning microscope analysis. (**a**–**c**) Control with addition of 10 μM (**b**) and 100 μM (**c**) of pargyline. No appreciable difference in 5-HT levels is seen in oocytes and granulosa cells. (**d**–**f**) Ovarian fragments with addition of 1 μM 5-HT after addition of 10 μM (**e**) and 100 μM (**f**) pargyline. 5-HT accumulation in oocytes does not change in the presence of pargyline, but the effect of a high 5-HT addition can be detected in granulosa cells. One of the growing follicles is outlined by a dotted line, the oocyte is marked with an asterisk, and the granulosa layer is marked with an arrowhead. Scale bar: 100 μm. (**g**) Quantitative analysis of 5-HT accumulation in oocytes (Oo) and granulosa cells (GC). *—indicates significant differences between the control and 5-HT groups at different pargyline concentrations (three-way comparison analysis ANOVA, *p* < 0.05).

In order to evaluate the effect of MAOI on exogenous 5-HT accumulation in the ovary, we performed experiments on a two-hour incubation of ovarian fragments with 5-HT and MAOI (pargyline). In these experiments, we used a model of 5-HT accumulation in the growing follicles of 14-day-old mice. At this stage, a large number of secondary follicles is presented in the ovary. Cryosections of ovarian fragments were immunostained by anti-5-HT antibodies. 5-HT accumulation in oocytes and granulosa cells of growing follicles was then analyzed on the confocal images (Figure 1).

The samples to which no 5-HT was added formed the control group (Figure 1a–c). The addition of pargyline (10 μM, 100 μM) alone did not significantly change the 5-HT level in the oocytes or follicle cells (Figure 1b,c).

As described in our previous work [8], the immunoreactivity of 5-HT in the oocytes of growing follicles is greatly increased in the presence of 5-HT (1 μM) (approximately 5-fold compared with the control group) (Figure 1d). The addition of pargyline (10 μM, 100 μM) had no effect on the 5-HT level in the oocytes (3.7- and 4.4-fold, respectively, compared with the control) (Figure 1e,f).

At the same time, a slight increase in the 5-HT level was detected in granulosa cells when 5-HT (1 μM) was added (by 1.9-fold compared with the control) (Figure 1d), but in the presence of pargyline (10 μM, 100 μM), an increase in the 5-HT level was detected (by 2.7-fold compared with the control group in both cases) (Figure 1e,f).

MAOI thus increases 5-HT accumulation in the granulosa cells of growing follicles, which may indicate MAO activity in these cells. In all likelihood, 5-HT uptake may be related to specific membrane transport driven by SERT.

### 2.2. Granulosa Cells Uptake 5-HT via SERT Activity

To study the accumulation and degradation of 5-HT in isolated follicular cells, we performed an experiment with a primary culture of mouse granulosa cells. We added pargyline to determine the activity of MAO and fluoxetine to see if SERT was also active in granulosa cells.

**Figure 2:**
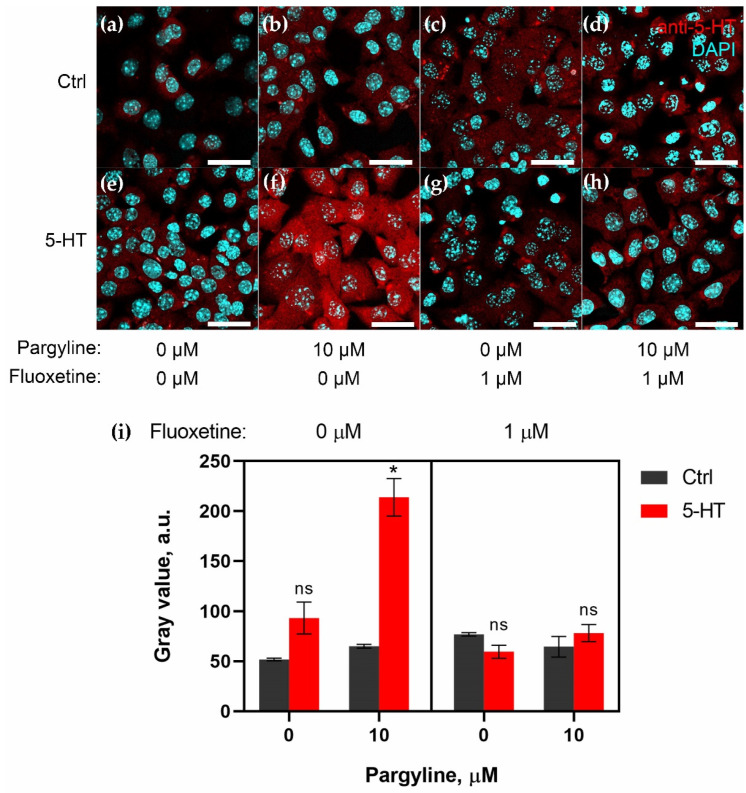
Effects of pargyline and fluoxetine on exogenous 5-HT accumulation in a primary culture of granulosa cells. (**a**–**h**) Immunocytochemical detection of 5-HT in granulosa cells after a two-hour incubation prepared by confocal laser scanning microscope analysis. (**a**–**d**) Control with addition of 10 μM pargyline and/or 1 μM fluoxetine. The difference in 5-HT levels is insignificant. (**e**–**h**) Granulosa cell culture with addition of 1 μM 5-HT after addition of 10 μM pargyline and/or 1 μM fluoxetine. The most remarkable effect of 5-HT accumulation is seen in the sample with the addition of 10 μM pargyline (**f**). The addition of 1 μM fluoxetine completely prevents this effect (**h**). Scale bar: 30 μm. (**i**) Quantitative analysis of 5-HT accumulation in granulosa cells. *—indicates significant differences between control and 5-HT groups using a triple ANOVA multiple comparison analysis, *p* < 0.05.

Cells were immunostained with anti-5-HT antibodies. We analyzed 5-HT uptake in granulosa cells on confocal images (Figure 2). Samples without added 5-HT were placed in the control group (Figure 2a–d). In samples to which only pargyline or/and fluoxetine was added, there was no significant enhancement (Figure 2b–d). The addition of 5-HT (1 μM) slightly increased 5-HT accumulation in the cytoplasm of the cells (by 1.8-fold, when compared with the control group) (Figure 2e). The effect of 5-HT accumulation was prevented by the addition of fluoxetine (Figure 2g). We saw the most remarkable effect in the sample in which pargyline (1 μM) was added before 5-HT (3.3-fold compared with control) (Figure 2f). When both fluoxetine and pargyline were added before 5-HT, there was no remarkable accumulation effect (Figure 2h).

Quantitative calculation of 5-HT accumulation in the granulosa cells of all experimental groups (Figure 2i) showed a significant increase in 5-HT levels in the sample to which both pargyline and 5-HT were added. A slight increase was observed in the group to which only 5-HT was added, but it was not significantly different from that of the control group. This result confirms the activity of MAO in this cell type. The fact that the addition of fluoxetine with pargyline before the addition of 5-HT prevented its accumulation also suggests that 5-HT uptake in granulosa cells is controlled by SERT.

### 2.3. Granulosa Cells of Growing Follicles Act as a Functional Barrier for 5-HT

SERT and MAO activity in granulosa cells may affect the ability of the oocyte to accumulate 5-HT. This cell layer acts as an obstacle between 5-HT in the blood and the oocyte as the main site of 5-HT accumulation in the ovary. To test this hypothesis, we compared the intensity of 5-HT accumulation in oocytes within growing follicles in ovarian tissue and in isolated oocytes. In this experiment, we sought to estimate the 5-HT concentration at which 5-HT does not accumulate in oocytes within the follicle (intact), but does accumulate in isolated oocytes (denuded). 5-HT concentrations were taken below the threshold at which appreciable accumulation of 5-HT in the ovary is observed: 10 nM, 25 nM, 50 nM, 75 nM, or 100 nM.

**Figure 3:**
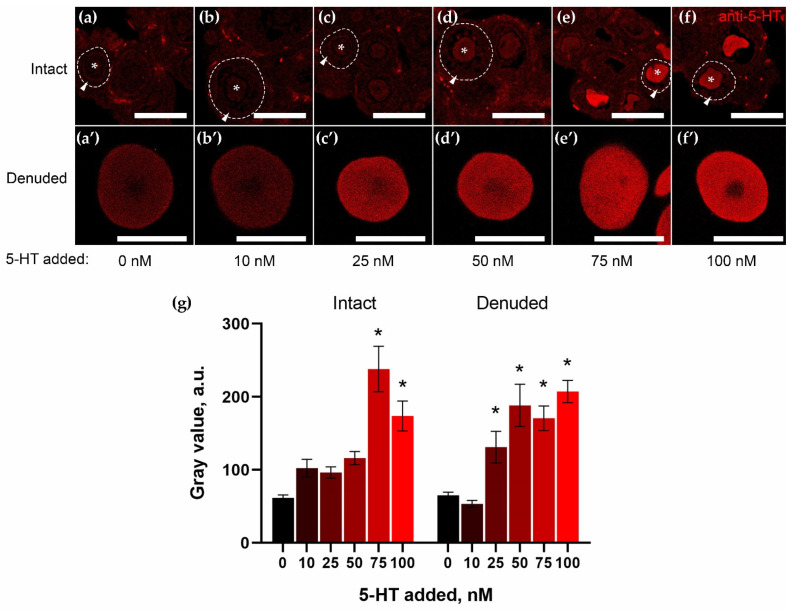
Concentration dependence of oocytes’ ability to accumulate exogenous serotonin within intact follicles or after their denudation. (**a**–**f**,**a**’–**f**’) Immunocytochemical detection of 5-HT in ovarian tissue (**a**–**f**) and denuded oocytes (**a**’–**f**’) after incubation with 0 nM (**a**,**a**’), 10 nM (**b**,**b**’), 25 nM (**c**,**c**’), 50 nM (**d**,**d**’), 75 nM (**e**,**e**’), and 100 nM (**f**,**f**’) 5-HT for 2 h. Prepared by confocal laser scanning microscope analysis. One of the growing follicles is outlined by a dotted line, the oocyte is marked with an asterisk, and the granulosa layer is marked with an arrowhead. Scale bar: (**a**–**f**) 100 μm, (**a’**–**f**’) 50 μm. (**g**) Quantitative analysis of 5-HT accumulation in intact and denuded oocytes. *—Shows significant differences between control (0 nM) and different 5-HT concentrations within groups of intact and denuded oocytes using one-way ANOVA multiple comparison analysis, *p* < 0.05.

After incubation with 5-HT for two hours, cryosections of ovarian fragments and isolated oocytes were immunostained with anti-5-HT antibodies and 5-HT uptake was analyzed (Figure 3). In intact follicles, there was a significant accumulation of 5-HT in oocytes when 75 nM 5-HT was added (Figure 3e,f). On the other hand, denuded oocytes significantly accumulated 5-HT when the concentration was 25 nM and higher (Figure 3c’–f’). These results indicate that the ability of oocytes to take up 5-HT increases in the absence of the granulosa cell barrier. This confirms that the granulosa cells surrounding the oocytes impede the accumulation of 5-HT in the oocytes through the activity of SERT and MAO-A.

## 3. Discussion

It is known that, in mammals, mature oocytes and preimplantation embryos express 5-HT receptors and are able to take up 5-HT from their environment [4,14]. Moreover, 5-HT is known to be one of the factors regulating the process of egg maturation in a variety of animal species [3,15,16,17,18,19,20,21,22,23]. 5-HT is detected at physiologically active concentrations in oocytes, cumulus cells [24], and follicular fluid [25].

Using various models, it has been shown that 5-HT is functionally active in the ovary and has a stimulatory effect on its function. Thus, 5-HT increases the synthesis of estradiol in the preovulatory follicles of hamsters and rats cultured in vitro [15,17]. This stimulatory effect of 5-HT is also observed in human granulosa cells, which is of particular interest [26,27]. Furthermore, in human antral follicles obtained using assisted reproductive technologies, 5-HT content in follicular fluid has been shown to correlate with both the morphological maturation of the oocyte and the success of the subsequent in vitro fertilization procedure [25].

The function of 5-HT in the ovary as a signaling molecule is ensured by the expression of a number of components of the serotonergic system, including appropriate synthetic enzymes, a vesicular transporter, several membrane 5-HT-receptors, as well as the main target of antidepressants, the 5-HT transporter SERT [4]. Expression of the *Sert* gene mRNA is detected in both follicular cells and oocytes [24]. In in vitro experiments, a pronounced specific membrane transport of 5-HT is observed in oocytes, the uptake of 5-HT in granulosa cells is noted, but is statistically not significant [8]. At the same time, fluoxetine affects cytosolic cAMP, ATP, and Ca^2+^ responses to forskolin, as well as the survival of human ovarian COV434 granulosa tumor cells, indicating SERT activity in these cells [28]. In a number of studies conducted in laboratory animals, antidepressants from the SSRI group have been shown to have negative effects on female reproductive function. For example, fluoxetine, when taken during prepuberty, impairs folliculogenesis in rats [29,30]. Fluoxetine ingestion in pregnant female rats leads to reproductive cycle disruptions, changes in the follicular pool, including an increase in ovarian apoptosis, and quantitative changes in the expression of genes that regulate 5-HT signaling and biological rhythms in offspring [31]. In adult mice that chronically consume paroxetine, suppression of estrogen synthesis is observed, as well as knockouts for the *Sert* gene, leading to obesity and the development of diabetes mellitus [32,33]. It is worth noting that these pathological conditions are risk factors for the development of female diseases, such as polycystic ovary syndrome [34].

A significant omission has been the lack of data on the functional activity of the enzymatic degradation system in the mammalian ovary. In this study, we have shown that both membrane transport and degradation of 5-HT occur in the granulosa cells of growing follicles. This fact explains the previous data indicating that the activity of the 5-HT transporter in the granulosa cells was masked by the high intensity of MAO: Most of the accumulated 5-HT was degraded soon after uptake.

Expression of *Maoa* mRNA was detected in mouse oocytes, as well as follicular cells [7]. Moreover, both *MAOA* and *MAOB* transcripts are detected in the RNA-seq data of human granulosa cells [35]. Monoamine oxidase activity also correlates with the estrous cycle in rats [36]. The activity of MAO in the different ovarian compartments has been poorly described. In the ovary of the pregnant rat, the most MAO-rich regions are the corpora lutea [37]. In our work, we have shown that inhibition of MAO in the ovary leads to a large increase in 5-HT accumulation in granulosa cells, but not in oocytes. Pargyline is considered as an MAO-B inhibitor, but it can also inhibit both MAO isoforms [38]. In our experiment, pargyline also altered 5-HT levels in tissues, especially in granulosa cells. It is known that MAO-B may also be involved in the degradation of 5-HT, but this reaction is considered to be very slow and ineffective. Though concentration of pargyline used in this work is enough to inhibit both MAO-A and MAO-B activity in the tissue [39], we consider that the high accumulation effect in the granulosa was achieved this particular way. Therefore, we suggest that MAO-A may be an active catalyst of 5-HT degradation in granulosa cells.

**Figure 4:**
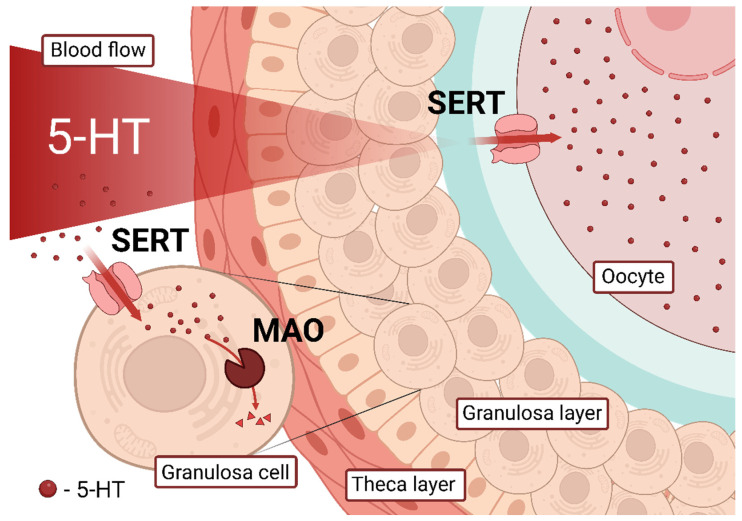
Granulosa cells form a functional barrier to 5-HT in the growing ovarian follicle. Maternal 5-HT circulating in the bloodstream is the main source of 5-HT in the ovary. At the same time, the growing oocyte is able to accumulate 5-HT with the help of the membrane transporter SERT. Between these two points lies a layer of granulosa cells that uptake 5-HT and degrade it with the help of MAO. This creates a functional barrier for 5-HT and isolates the growing oocytes from it until ovulation.

In this work, we have shown that granulosa cells alter 5-HT uptake in oocytes through accumulation and degradation. Given that oocytes have high 5-HT uptake activity, the role of follicular granulosa cells may be to capture and degrade external 5-HT to protect the growing oocyte from it, as a functional part of the so-called blood–follicle barrier (BFB) (Figure 4). According to the traditional view, the blood–follicle barrier consists of the vascular endothelium, the interstitium of the theca cells, the basement membrane of the granulosa cells, the granulosa membrane, and in the case of antral follicles, the follicular fluid of the antrum [40]. In antral follicles, this barrier is considered a “molecular sieve” because molecules less than 500 kDa in size can pass through unimpeded [41]. It is known that some molecules (~200 kDa) can pass through the BFB only after the LH increases as an ovulatory stimulus [42]. 5-HT is a very small molecule (176 Da) that can readily diffuse through the extracellular space into tissues, but due to its hydrophilic properties requires an active transport system to enter cells. In experiments with a range of low 5-HT concentrations, it was shown that uptake into isolated oocytes becomes remarkable with the addition of a lower amount of 5-HT than in oocytes from intact follicles. Our results suggest that active 5-HT accumulation and degradation systems in follicular granulosa cells may act not as a physical, but as a functional barrier to 5-HT uptake in follicles and may moderate 5-HT levels in oocytes.

We demonstrated a significant effect of 5-HT accumulation in oocytes enclosed by granulosa cells at a relatively low concentration of 75 nM. Presumably, in the event of an increase in 5-HT concentration in blood vessels, the function of the follicular 5-HT barrier may be disrupted and oocytes may receive additional exogenous 5-HT. An increase in bloodstream 5-HT levels has been demonstrated in several diseases, for example, primary pulmonary hypertension and inflammation [43]. Apparently, the barrier function of the BFB is disrupted just before ovulation by changes in the epithelial structure of the granulosa cells, the ingrowth of vessels and theca cells into the granulosa layer, and the invasion of immune cells into the follicle. Intrafollicular 5-HT levels increase due to immune cell activity, especially mast cells [44]. During ovulation, mature oocytes fall into the fallopian tubes, where 5-HT concentration is higher than in the ovary [45]. Therefore, the mechanism of 5-HT uptake during this period may have functional significance and influence the processes of early development.

The study of the functional activity of the components of the serotonergic system in ovarian cells and early embryos is of great interest in the context of the increasing popularity of the neurochemical treatment of mood disorders. Thus, antidepressants from the SSRI group, as well as MAO inhibitors, may have direct effects on ovarian function and on the course of oocyte maturation and preimplantation development. The long-term effects of such exposure still need to be clarified in further studies.

## 4. Materials and Methods

### 4.1. Experimental Animals and Chemicals

Female C57BL/6 mice from the Laboratory Animal Center of the Koltzov Institute for Developmental Biology RAS, were used for the animal experiments. Animals were kept under controlled conditions (22–24 °C and 14L:10D photoperiod). Mice were given ad libitum access to food and water.

The chemicals used in the study were 5-HT-creatinine sulfate (H7752 Merck KGaA, Darmstadt, Germany), pargyline hydrochloride (P8013 Merck KGaA, Darmstadt, Germany), and fluoxetine hydrochloride (PHR1394 Merck KGaA, Darmstadt, Germany). In all experiments, pargyline and/or fluoxetine were added 20 min before the addition of 5-HT.

### 4.2. Ovary Fragments and Denuded Oocytes Incubation Experiments

The ovaries of 14-day-old mice were placed on ice in Dulbecco’s PBS buffer (Biosera, Nuaille, France) and washed to remove blood. The ovaries were placed in pre-warmed Hank’s saline buffer (Biosera, Nuaille, France) and dissected into eight pieces each. The ovarian fragments were randomized and incubated for 2 h at 37 °C in four-well culture dishes (Thermo Fisher Scientific Inc., Waltham, MA, USA) containing 1 mL L15 medium (Thermo Fisher Scientific Inc., Waltham, MA, USA) supplemented with chemicals.

To isolate oocytes from growing follicles, ovaries were pre-incubated with 6 mM EGTA (Merck KGaA, Darmstadt, Germany) and 0.5 M sucrose (Merck KGaA, Darmstadt, Germany), and oocytes were released by rupturing the follicles with a 29G needle. After washing, the denuded oocytes were incubated in a chemical-enriched L15 medium for 2 h.

### 4.3. Granulosa Cells Incubation Experiments

Ovaries from PMSG-stimulated mice (Follimag (Mosagrogen, Moscow, Russia) – 5 IU, 48 h, subcutaneous) were preincubated with 6 mM EGTA and 0.5 M sucrose [46] and released by follicle puncture with a 29G needle. Cell clumps and oocytes were removed by filtering the cell suspensions through a 40-μm nylon cell strainer (BD Biosciences, Franklin Lakes, NJ, USA). Granulosa cell viability was determined by trypan blue exclusion. Granulosa cells (0.9 × 10^6^ per well in a 6-well plate) were plated overnight in DMEM/F-12 (Biolot, St. Petersburg, Russia) containing 10% FBS (Biosera, Nuaille, France) under a humidified atmosphere of 95% air and 5% CO_2_. Cells were washed with serum-free medium and treated with chemicals for 2 h.

### 4.4. Immunohistochemistry

For immunofluorescence, ovarian fragments, oocytes, or granulosa cells were fixed overnight in 4% paraformaldehyde (PFA) at 4 °C. 5-HT immunostaining of ovarian cryosections and an estimation of 5-HT immunoreactivity was performed as described in [8]. Briefly, samples were permeabilized by PBS (PanEco, Moscow, Russia) with 0.1% Triton X-100 (Merck KGaA, Darmstadt, Germany) (PBST), then blocked with 2% normal goat serum (Merck KGaA, Darmstadt, Germany), 1% BSA (Merck KGaA, Darmstadt, Germany), 0.1% cold fish skin gelatin (Merck KGaA, Darmstadt, Germany) in PBST, and stained with rabbit anti-5-HT antibody (S5545 Merck KGaA, Darmstadt, Germany – overnight, 4 °C) and goat anti-rabbit IgG antibody conjugated with CF 568 (SAB4600085 Merck KGaA, Darmstadt, Germany – 1 h, RT). In the case of granulosa cell culture, DNA was counterstained with DAPI (Merck KGaA, Darmstadt, Germany). Samples were then embedded in Mowiol for subsequent analysis with a microscope.

### 4.5. Image Analysis and Statistics

We analyzed the samples using a confocal laser scanning microscope Leica TCS SP5 (Leica Microsystems GmbH, Wetzlar, Germany). All samples in an experiment were analyzed with the same microscope and software characteristics: objectives, laser intensity, and detector sensitivity values. Photomicrographs were analyzed using FIJI software (open-source project). 5-HT levels were quantified based on fluorescence intensity using the mean gray level instrument for the regions of interest (oocytes or granulosa cells). Statistical analysis was performed using GraphPad Prism software (GraphPad Software, San Diego, CA, USA).

## Data Availability

Not applicable.

## References

[1] El-Merahbi R., Löffler M., Mayer A., Sumara G. (2015). The roles of peripheral serotonin in metabolic homeostasis. FEBS Lett..

[2] Spohn S.N., Mawe G.M. (2017). Non-conventional features of peripheral serotonin signalling—The gut and beyond. Nat. Rev. Gastroenterol. Hepatol..

[3] Sheng Y., Wang L., Liu X.S.J.S., Montplaisir V., Tiberi M., Baltz J.M., Liu X.S.J.S. (2005). A serotonin receptor antagonist induces oocyte maturation in both frogs and mice: Evidence that the same G protein ptor is responsible for maintaining meiosis arrest in both species. J. Cell. Physiol..

[4] Dubé F., Amireault P. (2007). Local serotonergic signaling in mammalian follicles, oocytes and early embryos. Life Sci..

[5] Shmukler Y.B., Nikishin D.A. (2022). Non-Neuronal Transmitter Systems in Bacteria, Non-Nervous Eukaryotes, and Invertebrate Embryos. Biomolecules.

[6] Perez V., Bel N., Celada P., Ortiz J., Alvarez E., Artigas F. (1998). Relationship Between Blood Serotonergic Variables, Melancholic Traits, and Response to Antidepressant Treatments. J. Clin. Psychopharmacol..

[7] Nikishin D.A., Khramova Y.V., Bagayeva T.S., Semenova M.L., Shmukler Y.B. (2018). Expression of Components of the Serotonergic System in Folliculogenesis and Preimplantation Development in Mice. Russ. J. Dev. Biol..

[8] Nikishin D.A., Alyoshina N.M., Semenova M.L., Shmukler Y.B. (2019). Analysis of Expression and Functional Activity of Aromatic L-Amino Acid Decarboxylase (DDC) and Serotonin Transporter (SERT) as Potential Sources of Serotonin in Mouse Ovary. Int. J. Mol. Sci..

[9] Yeung A.W.K., Georgieva M.G., Atanasov A.G., Tzvetkov N.T. (2019). Monoamine Oxidases (MAOs) as Privileged Molecular Targets in Neuroscience: Research Literature Analysis. Front. Mol. Neurosci..

[10] Chen K., Shih J.C. (1998). Monoamine oxidase A and B: Structure, function, and behavior. Adv. Pharmacol..

[11] Pickar D., Murphy D.L., Cohen R.M., Campbell I.C., Lipper S. (1982). Selective and nonselective monoamine oxidase inhibitors: Behavioral disturbances during their administration to depressed patients. Arch. Gen. Psychiatry.

[12] Allen D.L., Renner K.J., Luine V.N. (1993). Pargyline-induced increase in serotonin levels: Correlation with inhibition of lordosis in rats. Pharmacol. Biochem. Behav..

[13] Cecconi S., Ciccarelli C., Barberi M., Macchiarelli G., Canipari R. (2004). Granulosa cell-oocyte interactions. Eur. J. Obstet. Gynecol. Reprod. Biol..

[14] Čikoš Š., Fabian D.D., Makarevich A.V., Chrenek P., Koppel J., Cikos S., Fabian D.D., Makarevich A.V., Chrenek P., Koppel J. (2011). Biogenic monoamines in preimplantation development. Hum. Reprod..

[15] Terranova P.F., Uilenbroek J.T., Saville L., Horst D., Nakamura Y. (1990). Serotonin enhances oestradiol production by hamster preovulatory follicles in vitro: Effects of experimentally induced atresia. J. Endocrinol..

[16] Buznikov G.A., Nikitina L.A., Malchenko L.A., Trubnikova O.B., Galanov AYu (1993). The control of oocyte maturation in the starfish and amphibians by serotonin and its antagonists. Int. J. Dev. Biol..

[17] Tanaka E., Baba N., Toshida K., Suzuki K. (1993). Serotonin stimulates steroidogenesis in rat preovulatory follicles: Involvement of 5-HT2 receptor. Life Sci..

[18] Cerdà J., Subhedar N., Reich G., Wallace R.A., Selman K. (1998). Oocyte sensitivity to serotonergic regulation during the follicular cycle of the teleost Fundulus heteroclitus. Biol. Reprod..

[19] Zatylny C., Durantou F., Boucaud-Camou E., Henry J. (2000). Evidence of 5-hydroxytryptamine synthesis in the follicles of Sepia officinalis and direct involvement in the control of egg-laying. Mol. Reprod. Dev..

[20] Tinikul Y., Joffre Mercier A., Soonklang N., Sobhon P. (2008). Changes in the levels of serotonin and dopamine in the central nervous system and ovary, and their possible roles in the ovarian development in the giant freshwater prawn, Macrobrachium rosenbergii. Gen. Comp. Endocrinol..

[21] Stricker S.A., Smythe T.L. (2001). 5-HT causes an increase in cAMP that stimulates, rather than inhibits, oocyte maturation in marine nemertean worms. Development.

[22] Lister A., Regan C., Van Zwol J., Van Der Kraak G. (2009). Inhibition of egg production in zebrafish by fluoxetine and municipal effluents: A mechanistic evaluation. Aquat. Toxicol..

[23] Wang Q., He M. (2014). Molecular characterization and analysis of a putative 5-HT receptor involved in reproduction process of the pearl oyster Pinctada fucata. Gen. Comp. Endocrinol..

[24] Amireault P., Dubé F. (2005). Serotonin and its antidepressant-sensitive transport in mouse cumulus-oocyte complexes and early embryos. Biol. Reprod..

[25] Bòdis J., Bognàr Z., Hartmann G., Török A., Csaba I.F. (1992). Measurement of noradrenaline, dopamine and serotonin contents in follicular fluid of human graafian follicles after superovulation treatment. Gynecol. Obstet. Investig..

[26] Graveleau C., Paust H.J., Schmidt-Grimminger D., Mukhopadhyay A.K. (2000). Presence of a 5-HT7 receptor positively coupled to adenylate cyclase activation in human granulosa-lutein cells. J. Clin. Endocrinol. Metab..

[27] Koppan M., Bodis J., Verzar Z., Tinneberg H.-R., Torok A. (2004). Serotonin may alter the pattern of gonadotropin-induced progesterone release of human granulosa cells in superfusion system. Endocrine.

[28] Nguyen T.M.D., Klett D., Combarnous Y. (2021). Fluoxetine affects cytosolic cAMP, ATP, Ca 2+ responses to forskolin, and survival of human ovarian granulosa tumor COV434 cells. Korean J. Physiol. Pharmacol..

[29] Romero-Reyes J., Cárdenas M., Damián-Matsumura P., Domínguez R., Ayala M.E. (2016). Inhibition of serotonin reuptake in the prepubertal rat ovary by fluoxetine and effects on ovarian functions. Reprod. Toxicol..

[30] Ulker N., Yardimci A., Kaya Tektemur N., Colakoglu N., Ozcan M., Canpolat S., Kelestimur H. (2020). Chronic exposure to paroxetine or bupropion modulates the pubertal maturation and the reproductive system in female rats. Reprod. Biol..

[31] Moore C.J., DeLong N.E., Chan K.A., Holloway A.C., Petrik J.J., Sloboda D.M. (2015). Perinatal Administration of a Selective Serotonin Reuptake Inhibitor Induces Impairments in Reproductive Function and Follicular Dynamics in Female Rat Offspring. Reprod. Sci..

[32] Zha W., Ho H.T.B., Hu T., Hebert M.F., Wang J. (2017). Serotonin transporter deficiency drives estrogen-dependent obesity and glucose intolerance. Sci. Rep..

[33] Zha W., Hu T., Hebert M.F., Wang J. (2019). Effect of Pregnancy on Paroxetine-Induced Adiposity and Glucose Intolerance in Mice. J. Pharmacol. Exp. Ther..

[34] Rojas J., Chávez M., Olivar L., Rojas M., Morillo J., Mejías J., Calvo M., Bermúdez V. (2014). Polycystic ovary syndrome, insulin resistance, and obesity: Navigating the pathophysiologic labyrinth. Int. J. Reprod. Med..

[35] Zhang Y., Yan Z., Qin Q., Nisenblat V., Chang H.M., Yu Y., Wang T., Lu C., Yang M., Yang S. (2018). Transcriptome Landscape of Human Folliculogenesis Reveals Oocyte and Granulosa Cell Interactions. Mol. Cell.

[36] Holzbauer M., Youdim M.B. (1973). The oestrous cycle and monoamine oxidase activity. Br. J. Pharmacol..

[37] Mihalik J., Maslankova J., Hodorova I., Ferenc P., Rybarova S., Marekova M. (2011). Relationship between serotonin and norepinephrine levels and the preimplantation embryo development in rat. Bratisl. Lek. Listy.

[38] Cawthon R.M., Breakefield X.O. (1979). Differences in A and B forms of monoamine oxidase revealed by limited proteolysis and peptide mapping. Nature.

[39] Murphy D.L., Karoum F., Pickar D., Cohen R.M., Lipper S., Mellow A.M., Tariot P.N., Sunderland T. (1998). Differential trace amine alterations in individuals receiving acetylenic inhibitors of MAO-A (clorgyline) or MAO-B (selegiline and pargyline). J. Neural Transm. Suppl..

[40] Siu M.K.Y., Cheng C.Y. (2012). The blood-follicle barrier (BFB) in disease and in ovarian function. Adv. Exp. Med. Biol..

[41] Cran D.G., Moor R.M., Hay M.F. (1976). Permeability of ovarian follicles to electron-dense macromolecules. Acta Endocrinol. (Copenh).

[42] Hess K.A., Chen L., Larsen W.J. (1998). The ovarian blood follicle barrier is both charge- and size-selective in mice. Biol. Reprod..

[43] Kéreveur A., Callebert J., Humbert M., Hervé P., Simonneau G., Launay J.M., Drouet L. (2000). High plasma serotonin levels in primary pulmonary hypertension. Effect of long-term epoprostenol (prostacyclin) therapy. Arterioscler. Thromb. Vasc. Biol..

[44] Duffy D.M., Ko C., Jo M., Brannstrom M., Curry T.E. (2019). Ovulation: Parallels With Inflammatory Processes. Endocr. Rev..

[45] Amenta F., Vega J.A., Ricci A., Collier W.L. (1992). Localization of 5-hydroxytryptamine-like immunoreactive cells and nerve fibers in the rat female reproductive system. Anat. Rec..

[46] Sèdes L., Leclerc A., Moindjie H., Cate R.L., Picard J.Y., Clemente N.D., Jamin S.P. (2013). Anti-Müllerian Hormone Recruits BMPR-IA in Immature Granulosa Cells. PLoS ONE.

